# One out of ten: low sampling efficiency of cloth dragging challenges abundance estimates of questing ticks

**DOI:** 10.1007/s10493-020-00564-5

**Published:** 2020-10-31

**Authors:** Siiri Nyrhilä, Jani J. Sormunen, Satu Mäkelä, Ella Sippola, Eero J. Vesterinen, Tero Klemola

**Affiliations:** 1 Department of Biology, University of Turku, FI-20014 Turku, Finland; 2 Biodiversity Unit, University of Turku, Turku, Finland; 3 Department of Ecology, Swedish University of Agricultural Sciences, Uppsala, Sweden

**Keywords:** *Borrelia*, Cloth dragging, *Ixodes ricinus*, Tick-borne pathogen, Sampling efficiency

## Abstract

Hard ticks (Acari: Ixodidae) act as important vectors of zoonotic pathogens. For instance, *Borrelia burgdorferi* s.l. spirochetes pose a severe health risk as aetiological agents of Lyme borreliosis. Commonly, to study the abundance of questing (host-seeking) ticks, a 1 m^2^ piece of cloth is dragged over vegetation for a determined distance. Here, we designed a tick-sampling study to estimate the sampling efficiency of this standard method. We established 10 m dragging transects in a hemiboreal mixed forest patch in SW Finland for a 5-day monitoring period. Five of the transects were cloth-dragged 3× a day, whereas another five transects were dragged 6× a day in a manner that after each morning, midday and afternoon dragging, a second dragging was conducted on the same transect immediately. Captured *Ixodes ricinus* ticks were subsequently analysed for tick-borne pathogens. The initial population size of nymphal ticks on a transect was approximated by the accumulated nymph catch from the dragging sessions. The sampling efficiency of the cloth dragging was low, as a single dragging in a previously untouched vegetation strip always caught less than 12% (mean 6%) of the estimated population of active nymphs that were assumed to be questing during the study. Clear results were not found for daily activity rhythm, as ticks were caught in all daily dragging sessions. Approximately every third nymph and every second adult carried a pathogen, but nothing indicated that the occurrence of a pathogen affected the likelihood of the tick being caught by cloth dragging. Our results suggest that only a minority of active ticks can be caught by a single cloth dragging. The abundance estimates in many tick investigations might thus be downward biased.

**Electronic supplementary material:**

The online version of this article (10.1007/s10493-020-00564-5) contains supplementary material, which is available to authorized users.

## Introduction

Hard tick species (Acari: Ixodidae) act as vectors for several bacterial, viral and protozoan pathogens, causing public health risks (Pfäffle et al. 2013; Hai et al. 2014; Rizzoli et al. 2014; de la Fuente et al. 2017; Estrada-Peña et al. 2017). Exophilic tick species, such as the most extensively studied and medically important species in Europe, *Ixodes ricinus* (the castor bean or sheep tick), quest on vegetation for meals on mammalian, avian or other hosts in each developmental stage: larva, nymph and adult females (adult males of some species may also feed on blood but do not fully engorge). When not actively questing, these ticks spend long off-host periods in the soil or litter layer (Needham and Teel 1991; Hermann and Gern 2015). Studies have indicated that *I. ricinus* populations have strongly grown in number in many places over the past few decades, and they are predicted to increase further (Gray et al. 2009; Rizzoli et al. 2014; Sormunen 2018; Sormunen et al. 2020a). In addition, since their distribution range is expanding northwards with current climate warming (Jaenson et al. 2012; Medlock et al. 2013; Jore et al. 2014; Alkishe et al. 2017; Laaksonen et al. 2017), efficient methods for the estimation of local tick abundance are needed.

Hard ticks are often sampled by dragging a 1 m^2^ piece of cloth, usually a cotton sheet, over the vegetation (Li and Dunley 1998; Ginsberg and Ewing 1989; Mays et al. 2016; Estrada-Peña et al. 2017; Kjær et al. 2019). The cloth simulates a passing host for questing ticks. By their extended legs, ticks latch onto the sheet and are then relatively easily counted, picked up and stored for further analyses. However, only a fraction of individuals occupying the dragged area can be sampled by this method because at any given time, only an unknown fraction of individuals is questing for host animals on vegetation (Needham and Teel 1991). In addition, another unknown fraction of questing ticks does not latch onto the cloth or is brushed off before the cloth is checked by the operator (Li and Dunley 1998; Borgmann-Winter and Allen 2020). In addition, any individual dragging is affected by specific characteristics of the sampling time and season: weather, vegetation type and ontogeny, operator (e.g., speed of walking), dragging transect length, and life stage structure of the tick population (Milne 1943; Li and Dunley 1998; Jensen 2000; Dobson 2013).

For cloth-dragging results to be even remotely comparable among studies, dragging should be conducted under similar conditions and vegetation structures with standardized equipment, and the dragged distance or area must be reported (Dobson 2013; Estrada-Peña et al. 2013). When estimating tick density in a certain area, it is recommended that the dragging is repeated at regular intervals, e.g., every 3rd week, during the tick season (Dobson 2013). Although these demands could be met by a precise study design, the obtained density index might still significantly deviate from the ‘absolute’ density of host-seeking ticks because of the low sampling efficiency of any single cloth dragging over a uniquely selected strip of vegetation and because, depending on the ambient humidity, questing ticks must occasionally descend from vegetation into the moister conditions of the litter and soil (Needham and Teel 1991; Hermann and Gern 2015). When sampling ticks and estimating their abundance in an area, repetitions to increase the precision of the density index are often performed on adjacent sampling transects (e.g., Mays et al. 2016; Kjær et al. 2019; Sormunen et al. 2020b), instead of repeating the dragging on the very same transect, with the latter being the method used here.

Several studies have suggested that the presence of pathogens influences the questing behaviour and fitness of ticks (Lefcort and Durden 1996; Hermann and Gern 2015; de la Fuente et al. 2017). For instance, tick-borne encephalitis virus (TBEV; the causative agent of tick-borne encephalitis in humans) has been linked to faster crawling speed and increased resistance to tick-repellents in *I. ricinus* (Belova et al. 2012). Likewise, the presence of *Borrelia burgdorferi* s.l., i.e., bacterial agents of Lyme borreliosis, in *I. ricinus* has been linked to decreased moving activity and increased resistance to desiccation, potentially facilitating longer questing periods (Hermann and Gern 2010, 2012). Given that such behavioural associations exist, at least in laboratory experiments, the presence of different pathogens in ticks may also affect cloth-dragging catch and, consequently, abundance estimates of the ticks in nature.

By repeated cloth dragging on the same sampling transects over five consecutive days, we studied the efficiency of the standard cloth-dragging sampling method for *I. ricinus*. To our knowledge, this has not been done before in the typical hemiboreal mixed forest habitat of the tick in northern Europe (but see Jensen 2000). Concurrently, our study provided data on the daily activity rhythm of *I. ricinus*. Furthermore, by analyzing the most common tick-borne pathogens in collected ticks, we investigated, whether the presence of a pathogen affects the likelihood of the tick being sampled by cloth dragging.

## Materials and methods

### Study site

The field study was conducted at Kuuva (60°24′41″N, 22°06′53″E) on the island of Ruissalo (within the city of Turku), SW Finland, from Monday to Friday in the 2nd week of June 2019. Ruissalo was chosen because of its relatively high *I. ricinus* density (14.2 ticks per 100 m^2^ in 2017) (Klemola et al. 2019), which lessens the randomness of the sampling results. The dragging transects were situated in a clearing in the mixed forest. The understory consisted mainly of blueberry (*Vaccinium myrtillus*), and the dominant tree species was pine (*Pinus sylvestris*).

### Tick sampling

We established 10 permanent dragging transects (sized 1 × 10 m) altogether. They were arranged in pairs of two within a 1-ha area of the study site. In each of five pairs, there was one type A transect (individual transects are labelled A1–A5) that was sampled only once in a sampling session, whereas the type B transect (labelled B1–B5) of the pair belonged to the repeated dragging arrangement, in which the dragging was repeated (dragging B_second_) immediately (< 5 min) after the removal and storing of the ticks of the first dragging (dragging B_first_). The transect type (A or B) of two parallel transects (distance < 10 m) in a pair was randomized by flipping a coin. Prevailing temperature (range: 13.2–21.8 °C) and relative humidity (40–98%) data during the dragging session were obtained from the closest observation station (Artukainen, Turku; distance 6 km) of the Finnish Meteorological Institute. Daily temperature means varied between 14.9–18.5 °C, and accumulated rainfall was negligible (0.6 mm) during the five study days.

The same 1 m^2^ white cotton cloth was used for all the sampling. A metal chain was sewn into the trailing edge of the cloth to act as a weight to press down vegetation. The dragging was conducted at a slow walking pace along the transect, always in the same direction and by the same operator. The type A transects were dragged 3× a day, once in the morning (from 9 to 11 a.m.), once in midday (from 11 a.m. to 13 p.m.) and once in the afternoon (from 13 to 15 p.m.), during the five study days. Repeated transects (i.e., type B transects) were dragged 6× a day, as each morning, midday and afternoon dragging was repeated immediately to further assess the efficiency of the cloth-dragging method. Consequently, our study consisted of 225 draggings (75 on the A transects and 150 on the B transects) over the course of 15 sessions. After dragging the ticks were counted based on their developmental stages, identified to species morphologically, and stored in ethanol-filled Eppendorf tubes in a freezer (− 20 °C). Additional walking on the dragging transects was avoided during the study.

### Estimation of population size and sampling efficiency

Because our cloth dragging in the same sampling transects removed ticks and thereby caused a decline in the catch per dragging, it was possible to approximate the transect-specific (for 10 m^2^) initial population size of active ticks using the Leslie and Davis (1939) regression method (Krebs 1989; see Tälleklint-Eisen and Lane 2000 for *Ixodes*). By this method, the population size is estimated by first regressing the catch of a cloth dragging on the accumulated catch (i.e., numbers of removed ticks) on a transect and then extrapolating the obtained linear regression to the *x*-axis. This method, however, worked only for the nymphs because larval numbers did not decline, despite their repeated removal, on the transects (see Results) and because numbers of caught adults were too low for meaningful analyses (see Results). Although it was difficult to prove scientifically with the tick data, we assumed that other assumptions of the Leslie and Davis method were sufficiently, although not fully, met (Krebs 1989): (1) the population of active nymphs was (relatively) closed, i.e., no significant immigration, emigration, emergences or deaths occurred during the 5-day study, (2) the probability of each individual nymph being caught was relatively constant throughout the study, and (3) all individual nymphs on a transect had the same probability of being caught.

For each cloth-dragging transect (n = 10), we performed the Leslie and Davis regression and thereby estimated the initial population size of active nymphs, the latter determined here as nymphs that were assumed to be at least occasionally questing during a study (in dragging sessions from Monday morning to Friday afternoon). For the type B transects (n = 5) of the repeated dragging arrangement, we used only the nymph catch of the first dragging (dependent variable) of each morning, midday and afternoon dragging session, whereas the catch of removed nymphs (independent variable) also accumulated from the instantly repeated (second) draggings. Finally, we calculated the sampling efficiency of the single cloth dragging by dividing the nymph catch of the very first 10 m dragging (conducted on the morning of the first study day) by the estimated initial population size on a transect. At best, the obtained sampling efficiency was an approximation because both the first catch and accumulated sum of removed nymphs were based on relatively low numbers of nymphs on a short sampling transect.

### Pathogen analyses

In spring 2020, we screened the sampled nymphs and adult ticks for the presence of TBEV and the most significant bacterial and protozoan pathogens of human or veterinary importance. We ignored the larval developmental stage, as larvae carried very few pathogens in Ruissalo in an earlier study (Klemola et al. 2019). The screened bacterial pathogens included the Lyme borreliosis agent *B. burgdorferi* s.l. group (*B. afzelii*, *B. burgdorferi* s.s., *B. garinii*, *B. valaisiana* and some unconfirmed ones), a tick-borne relapsing fever spirochete *B. miyamotoi*, human granulocytic anaplasmosis agent *Anaplasma phagocytophilum*, spotted fever agents *Rickettsia* spp., *Neoehrlichia mikurensis* (neoehrlichiosis), *Bartonella* spp. (e.g., cat scratch disease) and *Francisella tularensis* (tularemia). Protozoan parasites *Babesia* spp. (babesiosis) were also screened.

Protocols for DNA and RNA extraction from the collected ticks, as well as real-time quantitative PCR (qPCR) assay protocols for detection of the pathogens, followed our previous works (Sormunen et al. 2016, 2018, 2020a, b; Laaksonen et al. 2017, 2018; Klemola et al. 2019) and are fully described in the supplement (Online Resource 1).

### Statistical analysis

We analysed tick abundance data from cloth dragging using the generalized linear mixed model (GLMM) approach (Stroup 2013). Unfortunately, the numbers of adult females and adult males (32 and 25 individuals caught, respectively) were too low for meaningful analyses. At first, the number of caught nymphs from a 10 m dragging was the response variable, and the sampling method [either the solitary dragging transect (A) and the first (B_first_) or the second (B_second_) dragging of the instantly repeated arrangement], sampling day (Mon–Fri) and sampling time (morning, midday or afternoon) with their potentially interesting two-way interactions were the fixed explanatory factors. From the final model, however, we removed the sampling method × sampling time interaction, as it was clearly unnecessary [deduced from the obtained low F value (< 1.0) and high P value (> 0.6)] to explain the variation in catch numbers. To account for the temporal and spatial arrangements, we set the sampling transect (A1–A5 and B1–B5) as a random intercept effect. Then, we ran an identical GLMM for numbers of larvae only (F value < 1.0 and P value > 0.6 for the removed sampling method × sampling time interaction). For both count data sets, we chose a negative binomial error distribution and log link function. For the results for fixed explanatory factors, we provide estimated marginal means (i.e., least-squares means in SAS) with their asymmetric 95% confidence intervals (95% CI). These estimates were back-transformed by the inverse link option to the original count data scale (Stroup 2013). Because a 10 m dragging was an observational unit (n = 225), multiplication of the model-derived estimate by a factor of 10 can be used to obtain the commonly reported density index (i.e., tick individuals per 100 m^2^) for each transect.

The same three fixed factors (i.e., sampling method, day and time) formed the binomial GLMM analysis (with logit link function) of the pathogen data to model the probability that a screened tick was positive for a pathogen. Here, interactions were omitted but the sampling transect was used again as a random intercept effect. Instead of reporting the modelling results for infrequently detected single pathogen species or genera, we focused on two pooled groups with sufficiently high prevalence for an interpretation; these groups included the detection of any pathogen and the detection of a pathogen belonging to the *B. burgdorferi* s.l. group.

All the GLMMs were run by the procedure GLIMMIX in SAS 9.4 statistical software (Stroup 2013). For appropriate F-tests, we adjusted standard errors and denominator degrees of freedom by the Kenward-Roger correction (Kenward and Roger 2009) as suggested (Stroup 2013).

## Results

### Ticks

During the 5-day study, we collected 508 *Ixodes ricinus* ticks. Other tick species were not caught. Nymphs (274 individuals; 54% of total) were caught most frequently, followed by larvae (177; 35%), adult females (32; 6%) and adult males (25; 5%). The exact numbers of life stages caught by transect, day and time are given in the supplement (Online Resource 2). The total length of the cloth dragging was 2 250 m, yielding a density index (mean ± SE, n = 225 drags) of 22.6 ± 1.5 and 12.2 ± 1.0 individuals per 100 m^2^ for all ticks and for nymphs only, respectively. These estimates are inherently downward biased, because we conducted removal samplings over a short time period.

**Fig. 1:**
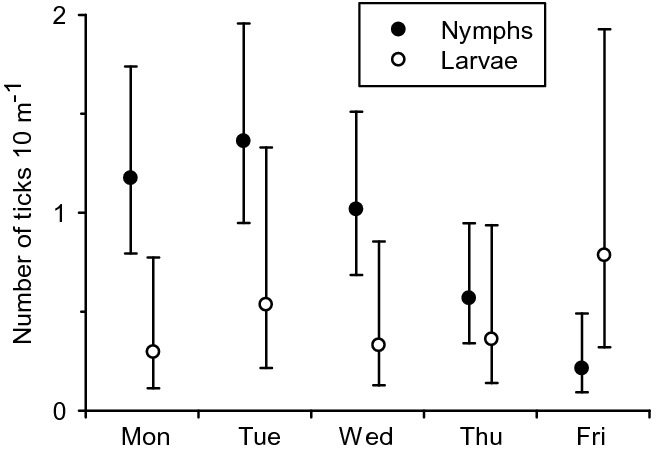
Model-derived least-squares mean (with 95% confidence intervals) estimates of caught nymphal and larval ticks (individuals per 10 m cloth dragging) on different sampling days. See Table 1 for GLMM statistics.

The GLMM for the number of caught nymphs revealed significantly fewer ticks in the second dragging of the instantly repeated arrangement compared to that under other types of dragging (effect ‘Sampling method’ in Table 1): the least-squares mean (95% CI) estimate of the 10 m cloth-dragging was 1.42 (0.93–2.16) nymphs for the type A dragging transect, 0.98 (0.64–1.50) nymphs for the transect B_first_, but only 0.27 (0.16–0.47) nymphs for the transect B_second_. Overall, the cloth dragging in the morning yielded more nymphs than that during midday or afternoon (‘Sampling time’ in Table 1): 1.16 (0.83–1.62) nymphs for the morning, 0.66 (0.45–0.95) nymphs for midday and 0.50 (0.30–0.82) nymphs for the afternoon. The highest daily number of nymphs was collected during the first 2 days of the study (Fig. 1), and thereafter, their numbers gradually declined towards the end of the study (‘Sampling day’ in Table 1, Fig. 1). The sampling day also had a marginally non-significant interactive effect with the sampling time (Table 1; Online Resource 2). The sampling transect as a random effect explained a significant amount of variation in nymph numbers [likelihood ratio test (LRT): χ^2^ = 13.3, df = 1, P = 0.0003].

**Table 1:** Test statistics of the fixed effects in GLMMs for the numbers of caught nymphs and larvae

Effect	Nymphs			Larvae		
	F	df	P	F	df	P
Sampling method	16.0	2,23.9	< 0.0001	4.3	2,9.0	0.049
Sampling time	10.7	2,200	< 0.0001	1.3	2,175.8	0.29
Sampling day	6.7	4,200	< 0.0001	2.3	4,200	0.06
Method × day	1.6	8,200	0.13	1.4	8,168.9	0.19
Time × day	2.9	8,187.2	0.063	1.6	8,160.4	0.13

The results for the numbers of larvae partly differed from those of the nymphs (Table 1). Daily numbers of larvae did not follow any pattern, and overall, larval data had much variation (Fig. 1, and 95% CI estimates for least-squares means given below). The highest numbers of larvae were caught on the last day of the study (Fig. 1). Similar to the nymphs, the lowest numbers of larvae [0.26 (0.08–0.83)] were caught on B_second_ transects compared to the type A [0.47 (0.15–1.48)] and B_first_ [0.66 (0.21–2.06)] transects. The sampling transect was also a significant random effect in the larval data (LRT: χ^2^ = 17.1, df = 1, P < 0.0001).

A further inspection of catch numbers solely from the type B dragging transects revealed that 78% (94 individuals) of nymphs and 75% (89 individuals) of larvae were already caught by the first dragging (B_first_) and only 22% (27 nymphs) and 25% (30 larvae) by the immediate subsequent dragging (B_second_). The catch numbers from the first and second draggings did not correlate with each other (Spearman correlation: nymphs, r_S_ = 0.13, P = 0.26; larvae, r_S_ = 0.15, P = 0.20; n = 75 dragging pairs).

**Fig. 2:**
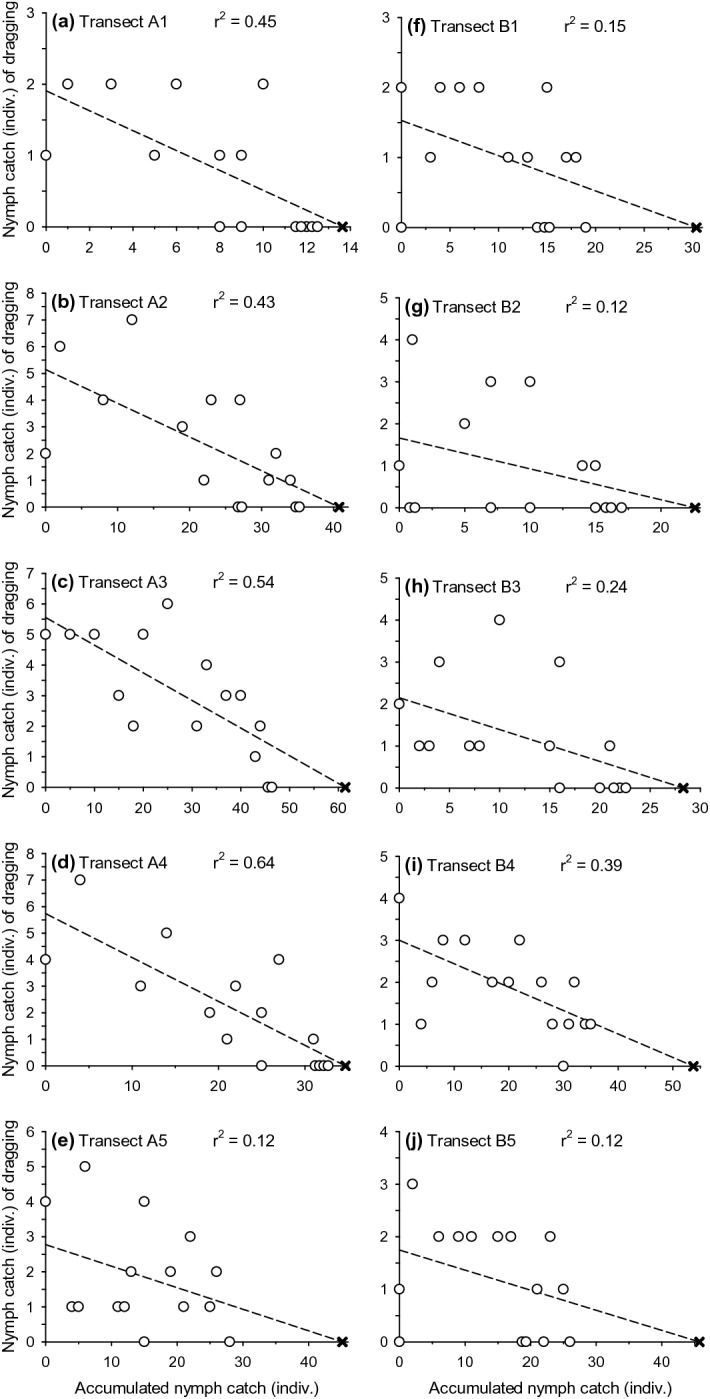
Leslie and Davis regressions to estimate the initial population size (N) of questing nymphs on 10 cloth-dragging transects (**a**–**j**). Symbols (×) point to the N estimates after extrapolation linear regressions to the x-axis (see Table 2). The coefficient of determination (r^2^) is provided on top of the regression panels. When originally overlapping, the circles are slightly skewed horizontally for illustrative purposes. Note also that scales of axes vary among panels.

The estimated sampling efficiency of a single cloth dragging in previously untouched vegetation remained at a low level regarding nymphal ticks (mean 6.0%, Table 2). However, the sampling efficiency for the nymphs seemed relatively constant among the transects (Table 2), and it did not correlate with the estimated initial population size (r = 0.11, P = 0.77; n = 10 transects). The estimation of the initial population size of nymphs is illustrated in Fig. 2.

**Table 2:** Sampling efficiency (%) estimates of the cloth-dragging method calculated as a percentage ratio of the catch of nymphal ticks in the first 10 m dragging divided by the estimated initial population size of questing nymphs on a transect

Transect	Catch (no. individuals)^a^	Estimated N^b^	First-drag catch (no. individuals)	Efficiency (%)
A1	12	13.6	1	7.3
A2	35	40.7	2	4.9
A3	46	61.4	5	8.1
A4	32	34.7	4	11.5
A5	28	45.1	4	8.9
B1	19	30.4	0	0.0
B2	17	22.6	1	4.4
B3	22	28.3	2	7.1
B4	37	53.8	4	7.4
B5	26	45.7	0	0.0
Mean ± SE (n = 10)	27.4 ± 3.3	37.6 ± 4.6	2.3 ± 0.5	6.0 ± 1.2

The population size (estimated N) of the nymphs was estimated using the Leslie and Davis regression for exploited populations (see Fig. 2)

^a^Total catch of nymphs from a transect (15 draggings on type A and 30 draggings on type B transects)

^b^Estimated, initial population size of questing nymphs on a transect

### Pathogens

From 331 (275 nymphs and 56 adults) screened ticks, 122 ticks (prevalence: 36.9%; 95% binomial CI: 31.7–42.3%) carried at least one pathogen (Table 3). The cooccurrence of two (or three in one case) pathogens was detected in 11 ticks (Table 3). The highest prevalence was found for bacteria belonging to the *B. burgdorferi* s.l. group. Furthermore, both bacterial pathogens, including *B. miyamotoi* (one detected from a nymph), *A. phagocytophilum*, *Rickettsia* spp. and *N. mikurensis*, and protozoan pathogens, including *Babesia* spp., were detected in the samples, although with low prevalence (Table 3). All screened ticks were negative for TBEV, *Bartonella* spp. and *Francisella tularensis*.

**Table 3:** Numbers (in parentheses, prevalence % [in brackets, 95% confidence interval]) of bacterial and protozoan (*Babesia* spp.) pathogens detected among *Ixodes ricinus* ticks that were cloth-dragged in Ruissalo (Turku, SW Finland) in early summer 2019

Stage	*Borrelia burgdorferi* s.l.	*B. miyamotoi*	*Rickettsia* spp.	*Anaplasma phagocytophilum*	*Neoehrlichia mikurensis*	*Babesia* spp.	Co-occurrence
Nymph (n = 275)	65^a^	1	9	17	3	6	7^b^
	(23.6 [18.7–29.1])	(0.4 [0.0–2.0])	(3.3 [1.5–6.1])	(6.2 [3.6–9.7])	(1.1 [0.2–3.2])	(2.2 [0.8–4.7])	(2.6 [1.0–5.2])
Adult (n = 56)	19^c^	0	3	4	4	2	4^d^
	(33.9 [21.8–47.8])	–	(5.4 [1.1–14.9])	(7.1 [2.0–17.3])	(7.1 [2.0–17.3])	(3.6 [0.4–12.3])	(7.1 [2.0–17.3])
Total (n = 331)	84	1	12	21	7	8	11
	(25.4 [20.8–30.4])	(0.3 [0.0–1.7])	(3.6 [1.9–6.3])	(6.3 [4.0–9.5])	(2.1 [0.9–4.3])	(2.4 [1.0–4.7])	(3.3 [1.7–5.9])

^a^Consisting of *B. afzelii* (4 detections), *B. burgdorferi* s.s. (4), *B. garinii* (38), a co-occurrence of *B. burgdorferi* s.s. and *B. garinii* (1), *B. valaisiana* (7) and unconfirmed (11)

^b^Co-occurrences in nymphs: *B. afzelii* and *N. mikurensis* (2 indiv.), *B. garinii* and *B. burgdorferi* s.s. and *Rickettsia* spp., *B. garinii* and *A. phagocytophilum* (2 indiv.), *Rickettsia* spp. and *A. phagocytophilum* (2 indiv.)

^c^Consisting of *B. burgdorferi* s.s. (2), *B. garinii* (8), *B. valaisiana* (4) and unconfirmed (5)

^d^Co-occurrences in adults: *B. garinii* and *N. mikurensis* (2 indiv.), *B. valaisiana* and *Rickettsia* spp. and *N. mikurensis* and *Babesia* spp.

According to the GLMM for the pooled nymphs and adults (to ensure larger sample size), the probability of detecting any kind of pathogen was not related to when the tick was caught or the sampling arrangement (sampling method: F_2,20.5_ = 1.53, P = 0.24; sampling time: F_2,322_ = 0.51, P = 0.60; sampling day: F_4,322_ = 0.12, P = 0.98; estimated least-squares means of the factors are given in Online Resource 3). The model for the *B. burgdorferi* s.l. group separately resulted in a qualitatively similar outcome (sampling method: F_2,18.7_ = 1.74, P = 0.20; sampling time: F_2,322_ = 0.51, P = 0.60; sampling day: F_4,322_ = 0.50, P = 0.74; Online Resource 3). The sampling transect did not explain the variation as a random effect in these two models (LRT: χ^2^ = 1.7, df = 1, P = 0.19 and χ^2^ = 0.6, df = 1, P = 0.43, respectively). Any further analyses solely for the nymphal stage (results not shown) or for rarer pathogens (results not shown) did not indicate that the questing behaviour of ticks was influenced by the occurrence of a pathogen.

## Discussion

Because standard cloth dragging is logistically easy, cost-effective and comparatively easy to standardize among field workers and across studies, it is commonly used and certainly an appropriate method for collecting a large number of ticks (Estrada-Peña et al. 2013; Kjær et al. 2019), e.g., for pathogen analyses (Sormunen et al. 2018; Kjær et al. 2020). However, it seemed here to be a rather inefficient method for estimating the ‘absolute’ abundance of actively questing ticks. Our results suggest that any single dragging caught only a minority of ticks that were questing during our short-term study. We assumed that the ‘population’ of active nymphs on a transect consisted of a determined number of ticks because our removal sampling decreased the nymph catch towards the end of the study. Nevertheless, an unknown fraction of the total tick population was in the inactive stage, probably throughout the whole study, and obtaining an abundance estimate for that fraction is a very challenging task.

Variation in the sampling efficiency was, however, small among our sampling transects, suggesting that the cloth-dragging method may generally yield a feasible relative density index, for example, for among-site comparisons. Specifically, a sufficiently unbiased index of relative tick density can be obtained, even if a small but constant fraction of the tick population is sampled at a time, if cloth-dragging transects are adequately replicated for precision (Li and Dunley 1998). Accordingly, we strongly advocate that tick density indices are routinely supplemented with a standard error (or 95% CI) estimate, which is attained by replicating individual sampling transects (e.g., of 10–50 m each) in a study area, instead of reporting only total numbers of ticks per total dragging distance. This is important because empirically based predictions for future tick population dynamics, and thereby evaluations of public health risks, cannot be based on vague density estimations by single dragging but warrant repetition both in time (e.g., every 3rd week; Dobson 2013) and space, as suggested here. Nevertheless, in type B dragging transects, approximately a quarter of nymphs and larvae were caught only by the second dragging of the session without obvious correlation to the catch number of the first dragging on the same transect. This result also indicates a moderate role of randomness in relation to how questing ticks latch onto the cloth during dragging.

Interestingly, our averaged sampling efficiency estimate (6% for nymphs) for the first dragging in untouched vegetation concurred well with earlier efficiency estimates conducted for *I. scapularis* (6.3%) in New York State (Daniels et al. 2000) and for *I. pacificus* (5.9%) in California (Tälleklint-Eisen and Lane 2000). One reason for our low sampling efficiency might be the blueberry-dominated understory that offers multiple layers for tick to questing. During sampling, the cloth swept over the upper layer of (woody) dwarf shrubs and did not necessarily reach the ground layer. Consequently, sampling efficiency might differ greatly in leaf litter or in flexible, grass-dominated understory vegetation, where the cloth reaches the ground better. Our results, however, are in disagreement with those reported by Jensen (2000), who suggested, by cloth dragging over three consecutive days in different habitats in Denmark, high sampling efficiency for the *I. ricinus* nymphs even when the vegetation was high and dense. As far as we are aware, no other sampling efficiency estimates have been made for *I. ricinus* in hemiboreal or boreal biotopes, where these ticks have become abundant in many kinds of deciduous, mixed and coniferous forest types, in addition to pastures, meadows and coastlines (Sormunen et al. 2016, 2020a; Estrada-Peña et al. 2017; Sormunen 2018; Kjær et al. 2019). This gap calls for research on tick sampling methodology in different vegetation types because standard cloth dragging has been an overriding method in *Ixodes* studies, especially in those conducted in Europe.

Had the sampling efficiency of the cloth-dragging method been higher, we would have expected our removal sampling to have emptied the transects of ticks during the last couple days of the study. However, as seen (Fig. 1, Online Resource 2), even 15 or 30 draggings did not deplete the sampling transects from nymphs, let alone larval ticks. Indeed, our results for larvae differed from those of nymphs. It might be that questing larvae stayed at lower levels of vegetation and were thereby haphazardly caught by the cloth (Mejlon and Jaenson 1997). In addition, tiny larvae are less mobile than older ticks and are spatially aggregated with the egg clusters oviposited by their mothers (Hauck et al. 2020). For these reasons, we did not obtain a sampling efficiency estimate for larvae at all. We suggest that density indices of larvae should be interpreted with caution, especially if they are based on low numbers of replicates.

We found a difference in catch numbers of nymphs attributed to the time of the day during the sampling. Overall, the morning dragging yielded more ticks than those conducted at midday or afternoon. However, our setup does not allow a firm interpretation because it may well be that a longer interval before the morning dragging explains the results by providing ticks more time to re-settle and attain a suitable questing position on the vegetation. Night-time mobility also reduces the risk of desiccation (Lees and Milne 1951; Perret et al. 2003). During the study in early summer, however, ambient temperature and relative humidity were favourable for ticks to quest throughout the day, and, as shown by the results (Table 2; Online Resource 2), the ticks were not inactive during midday or afternoon.

Gherman et al. (2012) increased the sampling efficiency of *I. ricinus* by releasing carbon dioxide (CO_2_) from a cylinder to the cloth. The usage of dry ice or other CO_2_ bait traps, has also been used in tick sampling with favourable results (Gray 1985; Ginsberg and Ewing 1989; Schulze et al. 1997; Mays et al. 2016). It remains to be studied, however, whether these modifications could sufficiently improve the sampling efficiency so that they could be logistically feasible and cost-effective in every-day tick research settings. The use of different modifications may also further limit the comparability among studies. Moreover, the target tick species, its developmental stage and vegetation type should always be considered when choosing the sampling method for ticks (Gherman et al. 2012; Mays et al. 2016).

In the current study, we detected the pathogenic species or genera, apart from TBEV, that are typically found in *I. ricinus* in the SW Finnish archipelago and elsewhere in southern Finland (Sormunen et al. 2016, 2018, 2020a, b; Laaksonen et al. 2017, 2018; Klemola et al. 2019). Pathogen prevalence in this study also corresponded well to earlier findings. Although detected in ticks elsewhere (Hai et al. 2014), we have not yet, despite screening thousands of ticks, found *Bartonella* spp. or *Francisella tularensis* in Finnish ticks (Sormunen et al. 2016, 2018, 2020a, b; Laaksonen et al. 2017, 2018; Klemola et al. 2019 and unpubl. data). Parasites, in general, are expected to maximize their transmission cycle by manipulating the behaviour of their hosts and vectors (Poulin 2010) and the ability of pathogenic microorganisms (e.g., *B. burgdorferi* s.l.) to modify the behaviour of ticks is accordingly recognized (Herrmann and Gern 2015). However, in the current field study, we did not find any evidence that pathogen-positive ticks would have been more active (or inactive) than non-infected ticks regarding questing and latching onto the cloth simulating a passing host animal. The probability of pathogen detection did not vary across the study days and sampling times (Online Resource 3). Unfortunately, the sample size of screened ticks and the low number of positive detections did not allow statistical analyses of all pathogen species separately.

Finally, we would like to remind researchers to recognize the fact that commonly used tick sampling methods from the field-layer vegetation catch only questing ticks (Mays et al. 2016) and that most published sampling efficiency estimates have indicated relatively poor catchability with any single attempt to sample these ticks. Thus, the absolute tick density on a given area must always be higher than the standard density estimate calculated for that area. Our rough sampling efficiency estimate (5–10%) for cloth dragging provides a correction factor for *I. ricinus* in hemiboreal mixed forests. When investigating distribution ranges, our results imply that the tick population is already very likely established when a researcher gets the first catch by cloth dragging in a new area.

## Electronic supplementary material

Below is the link to the electronic supplementary material.

Supplementary file1 (DOCX 31 kb)

Supplementary file2 (DOCX 82 kb)

Supplementary file3 (DOCX 18 kb)

## Data Availability

The datasets generated and analysed during the current study are available from the corresponding author on reasonable request.
