# Supplementary material for: One out of ten: low sampling efficiency of cloth dragging challenges abundance estimates of questing ticks
Source: Exp Appl Acarol. 2020 Oct 31;82(4):571–85. doi: 10.1007/s10493-020-00564-5 (PMC7686165; doi:10.1007/s10493-020-00564-5)
Supplement: Supplementary file 1 — Supplementary file1 (DOCX 31 kb) [file 10493_2020_564_MOESM1_ESM.docx]

*Online Resource 1*

**One out of ten: low sampling efficiency of cloth dragging challenges abundance estimates of questing ticks**

Siiri Nyrhilä^1^, Jani J. Sormunen^1,2^, Satu Mäkelä^1^, Ella Sippola^1,2^, Eero J. Vesterinen^2,3^ & Tero Klemola^1^

^1^ Department of Biology, University of Turku, Finland

^2^ Biodiversity Unit, University of Turku, Finland

^3^ Department of Ecology, Swedish University of Agricultural Sciences, Uppsala, Sweden

Corresponding author:

Tero Klemola

Address: Department of Biology, University of Turku, FI-20014 Turku, Finland

Tel.: +358 29 4504216

E-mail: [tero.klemola@utu.fi](mailto:tero.klemola@utu.fi)

*Supplementary material*

**Laboratory methods for detecting tick-borne pathogens**

DNA and RNA were extracted from the (frozen) tick samples using NucleoSpin® 96 RNA – kits, and RNA/DNA buffer sets (Macherey-Nagel, Germany), following the kit protocols (96 RNA Kit: Rev. 06/December 2018; RNA/DNA buffer set: Rev. 10/January 2018). DNA extracts were stored at -20 °C and RNA extracts were stored at -80 °C for later use.

DNA samples were screened for bacterial pathogens *Borrelia burgdorferi* sensu lato (including separate analyses for *B. afzelii, B. burgdorferi* s.s., *B.* garinii and *B. valaisiana*), *Borrelia miyamotoi*, *Anaplasma phagocytophilum*, *Rickettsia* spp., *Neoehrlichia mikurensis*, *Francisella tularensis* and *Bartonella* spp., and for protozoan parasites *Babesia* spp. Furthermore, RNA samples were screened for tick-borne encephalitis virus (TBEV). Analyses regarding *Borrelia* were carried out on individual DNA samples. For the screening of all other pathogens, samples were analyzed in pools (12 samples per pool, 5 μl of each sample) due to low expected prevalence. Individual samples from a pool found positive were subsequently re-analyzed separately. The primers used for each pathogen are reported in Table S1.

Real-time quantitative PCR (henceforth abbreviated qPCR) assays were carried out using SensiFAST™ Probe Lo-ROX Kit (for DNA) and SensiFAST™ Probe Lo-ROX One-Step Kit (for RNA) (Bioline, Germany). All DNA/RNA samples were analyzed in two replicate reactions carried out on 96 or 384-well plates. At least two blank water samples were used as negative controls in each assay. Samples were considered positive when successful amplification was detected in both replicate reactions or in two consecutive assays. Assay protocols and mastermix contents are given in Table S2.

For the qPCR analysis of *B. afzelii*, *B. burgdorferi* s.s., *B. garinii*, *B. valaisiana* and *B. miyamotoi* we used primers and probes specific to each genospecies (Table S1). These analyses were performed as duplex and multiplex assays: a duplex for *B. garinii* and *B. miyamotoi*, and a multiplex for *B. afzelii, B. burgdorferi* s.s. and *B. valaisiana*. Assays were carried out in 8 μl and 11 μl reaction volume, containing 2 μl of DNA extract. Laboratory-grown strains of *B. afzelii*, *B. burgdorferi* s.s. and *B. garinii* were used as positive controls for the respective pathogens, whereas for *B. valaisiana* and *B. miyamotoi*, we used samples previously identified via qPCR analysis. The thermal cycling profile used for analyses was 95°C for 5 minutes, then 50 cycles of 95°C for 10 sec and 60°C for 30 sec.

**Table S1.** Primers and probes used in tick-borne pathogen screening

––––––––––––––––––––––––––––––––––––––––––––––––––––––––––––––––––––––––––––––––––––––––––––––––––––––––––––––––––––––––––––––––––––

Primer/probe name Primer/probe target 5’ → 3’ Reference

––––––––––––––––––––––––––––––––––––––––––––––––––––––––––––––––––––––––––––––––––––––––––––––––––––––––––––––––––––––––––––––––––––

Bb23Sf *B. burgdorferi* 23S RNA CGAGTCTTAAAAGGGCGATTTAGT Courtney et al. 2004

Bb23Sr *B. burgdorferi* 23S RNA GCTTCAGCCTGGCCATAAATAG

Bb23Sp *B. burgdorferi* 23S RNA [FAM]-AGATGTGGTAGACCCGAAGCCGAGTG-[BHQ1]

Baf-RecA-F *B. afzelii recA* AGTCAGCCTGATACCGGAGA Tveten 2013

Baf-RecA-R *B. afzelii recA* ATTTTGGGGTCAAAGCTGCC

Baf-RecA-P *B. afzelii recA* [FAM]-TGCCGAACATTTAATTAGAAG-[BHQ1]

Bbss-RecA-F *B. burgdorferi* s.s. *recA* CCTGATACCGGAGAGCAAGC Tveten 2013

Bbss-RecA-R *B. burgdorferi* s.s. *recA* GGGGTTAAAGCCGCTACAGA

Bbss-RecA-P *B. burgdorferi* s.s. *recA* [HEX]-TTGCTGAGCATTTAATCAGAA-[BHQ1]

Bga-RecA-F *B. garinii recA* ATGCAAAAGCTTTGGGGGTT Tveten 2013

Bga-RecA-R *B. garinii recA* AGGGGTTAAAGCTGCTACAGA

Bga-RecA-P *B. garinii recA* [HEX]-TTGCCGAACATTTAATCAGAA-[BHQ1]

Bva-RecA-F *B. valaisiana recA* TGGTCCTGAGTCGTCTGGTA Tveten 2013

Bva-RecA-R *B. valaisiana recA* CTTGCTCTCCGGTGTCAGG

Bva-RecA-P *B. valaisiana recA* [Cy5]-AGGTTCAAAAAGAAGGTGGTAT-[BHQ2]

Bmi-F *B. miyamotoi glpQ* CACGACCCAGAAATTGACACA Vayssier-Taussat et al. 2013

Bmi-R *B. miyamotoi glpQ* GTGTGAAGTCAGTGGCGTAAT

Bmi-P *B. miyamotoi glpQ* [FAM]-TCGTCCGTTTTCTCTAGCTCGATTGGG-[BHQ1]

Bart-ssRA-F *Bartonella ssRa* GCTATGGTAATAAATGGACAATGAAATAA Diaz et al. 2012

Bart-ssRA-R *Bartonella ssRa* GCTTCTGTTGCCAGGTG

Bart-ssRA-P *Bartonella ssRa* [FAM]-ACCCCGCTTAAACCTGCGACG-[BHQ1]

Rspp-F *Rickettsia gltA* GAGAGAAAATTATATCCAAATGTTGAT Labruna et al. 2004

Rspp-R *Rickettsia gltA* AGGGTCTTCGTGCATTTCTT

Rspp-P *Rickettsia gltA* [CY5]-CATTGTGCCATCCAGCCTACGGT-[BHQ3]

CNeGroEL-F *N. mikurensis* groEL CCTTGAAAATATAGCAAGATCAGGTAG Jahfari et al. 2012

CNeGroEL-R *N. mikurensis* groEL CCACCACGTAACTTATTTAGCACTAAAG

CNeGroEL-P *N. mikurensis* groEL [FAM]-CCTCTACTAATTATTGCWGAAGATGTAGAAGGTGAAGC-[BHQ1]

*Table S1 continues*

––––––––––––––––––––––––––––––––––––––––––––––––––––––––––––––––––––––––––––––––––––––––––––––––––––––––––––––––––––––––––––––––––––

Primer/probe name Primer/probe target 5’ → 3’ Reference

––––––––––––––––––––––––––––––––––––––––––––––––––––––––––––––––––––––––––––––––––––––––––––––––––––––––––––––––––––––––––––––––––––

ApMSP2F *A. phagocytophilum Msp2* ATGGAAGGTAGTGTTGGTTATGGTATT Courtney et al. 2004

ApMSP2R *A. phagocytophilum Msp2* TTGGTCTTGAAGCGCTCGTA

ApMSP2P *A. phagocytophilum Msp2* [CY5]-TGGTGCCAGGGTTGAGCTTGAGATTG-[BBQ650]

Bab18S-F *Babesia* 18S rRNA CAGCTTGACGGTAGGGTATTGG Radzijevskaja et al. 2008

Bab18S-R *Babesia* 18S rRNA TCGAACCCTAATTCCCCGTTA

Bab18S-P *Babesia* 18S rRNA [HEX]-CGAGGCAGCAACGG-[BHQ1]

TBE2-F TBEV non-struct. prot. 5 TGGGCGGTTCTTGTTCTCC Brinkley et al. 2008

TBE2-R TBEV non-struct. prot. 5 TCACACATCACCTCCTTGTCAGA

TBE2-P TBEV non-struct. prot. 5 [HEX]-CTGAGCCACCATCACCCAGACACAG-BHQ1

FTu23-F *F. tularensis* 23Kda TGAGATGATAACAAGACAACAGGTAAC Skottman et al. 2007

FTu23-R *F. tularensis* 23Kda GGATGAGATCCTATACATGCAGTAGGA

FTu23-P *F. tularensis* 23Kda [HEX]-CCATTCATGTGAGAACTG-[BHQ1]

––––––––––––––––––––––––––––––––––––––––––––––––––––––––––––––––––––––––––––––––––––––––––––––––––––––––––––––––––––––––––––––––––––

**Table S2.** Mastermix contents for qPCR screening of pathogens

––––––––––––––––––––––––––––––––––––––––––––––––––––––––––––––––––––––––––––––––––––––––––––––––––––––––––––––––––––––––––––––––––––

qPCR target Single/Duplex/ Sample(s) Volume Probe mix^a^ ddH_2_O Forward/reverse Probe DNA/RNA

Multiplex primer concentration^b^ concentration^b^ template

––––––––––––––––––––––––––––––––––––––––––––––––––––––––––––––––––––––––––––––––––––––––––––––––––––––––––––––––––––––––––––––––––––

*B. burgdorferi* s.l. Single Single 5 µl 2.5 µl 1.25 µl 200 nM 100 nM 1 µl

*Rickettsia* spp. Multiplex Pooled 11 µl 5.5 µl 0.3 µl 300 nM 150 nM 3 µl

*Bartonella* spp. Multiplex 200 nM 100 nM

*Francisella* spp. Multiplex 300 nM 150 nM

*Rickettsia* spp. Single Single 5 µl 2.5 µl 1.25 µl 200 nM 100 nM 1 µl

*A. phagocytophilum* Multiplex Pooled 11 µl 5.5 µl 0.75 µl 400 nM 200 nM 2 µl

*Babesia* spp. Multiplex 400 nM 200 nm

*N. mikurensis* Multiplex 200 nM 100 nM

*A. phagocytophilum* Single Single 5 µl 2.5 µl 1.25 µl 200 nM 100 nM 1 µl

*Babesia* spp. Single Single 5 µl 2.5 µl 1.25 µl 200 nM 100 nM 1 µl

*N. mikurensis* Single Single 5 µl 2.5 µl 1.25 µl 200 nM 100 nM 1 µl

*B. afzelii* Multiplex Single 11 µl 5.5 µl 1.025 µl 300 nM 150 nM 2 µl

*B. burgdorferi* s.s. Multiplex 300 nM 150 nM

*B. valaisiana* Multiplex 300 nM 150 nM

*B. afzelii* Single Single 5 µl 2.5 µl 1.25 µl 200 nM 100 nM 1 µl

*B. burgdorferi* s.s. Single Single 5 µl 2.5 µl 1.25 µl 200 nM 100 nM 1 µl

*B. valaisiana* Single Single 5 µl 2.5 µl 1.25 µl 200 nM 100 nM 1 µl

*B. garinii* Duplex Single 8 µl 4.0 µl 1.0 µl 300 nM 150 nM 2 µl

*B. miyamotoi* Duplex 200 nM 200 nM

*B. garinii* Single Single 5 µl 2.5 µl 1.25 µl 200 nM 100 nM 2 µl

*B. miyamotoi* Single Single 5 µl 2.5 µl 1.25 µl 200 nM 100 nM 1 µl

TBEV Single Pooled 8 µl 4 µl 0.13 µl 400 nM 100 nM 3 µl

TBEV Single Single 5 µl 2.5 µl - 400 nM 100 nM 1,9 µl

––––––––––––––––––––––––––––––––––––––––––––––––––––––––––––––––––––––––––––––––––––––––––––––––––––––––––––––––––––––––––––––––––––

^a^ For DNA samples: SensiFAST™ Probe Lo-ROX Kit; For RNA samples: SensiFAST™ Probe Lo-Rox One-Step Kit

^b^ See Table S1 for primer/probe sequences

**References**

Brinkley C, Nolskog P, Golovljova I, Lundkvist Å, Bergström T (2008) Tick-borne encephalitis virus natural foci emerge in western Sweden. Int J Med Microbiol 298 (S1):73–80. <https://doi.org/10.1016/j.ijmm.2007.12.005>

Courtney JW, Kostelnik LM, Zeidner NS, Massung RF (2004) Multiplex real-time PCR for detection of *Anaplasma phagocytophilum* and *Borrelia burgdorferi*. J Clin Microbiol 42:3164–3168.

<https://doi.org/10.1128/JCM.42.7.3164-3168.2004>

Diaz MH, Bai Y, Malania L, Winchell JM, Kosoy MY (2012) Development of a novel genus-specific real-time PCR assay for detection and differentiation of *Bartonella* species and genotypes. J Clin Microbiol 50:1645–1649.

<https://doi.org/10.1128/JCM.06621-11>

Jahfari S, Fonville M, Hengeveld P, Reusken C, Scholte E-J, Takken W, Heyman P, Medlock JM, Heylen D, Kleve J, Sprong H (2012) Prevalence of *Neoehrlichia mikurensis* in ticks and rodents from North-west Europe. Parasites Vectors 5:74. <https://doi.org/10.1186/1756-3305-5-74>

Labruna MB, Whitworth T, Horta MC, Bouyer DH, McBride JW, Pinter A, Popov V, Gennari SM, Walker DH (2004) *Rickettsia* species infecting *Amblyomma cooperi* ticks from an area in the state of São Paulo, Brazil, where Brazilian spotted fever is endemic. J Clin Microbiol 42:90–98. <https://doi.org/10.1128/JCM.42.1.90-98.2004>

Radzijevskaja J, Paulauskas A, Rosef O (2008) Prevalence of *Anaplasma phagocytophilum* and *Babesia divergens* in *Ixodes ricinus* ticks from Lithuania and Norway. Int J Med Microbiol. 298: 218–221.

<https://doi.org/10.1016/j.ijmm.2008.01.008>

Skottman T, Piiparinen H, Hyytiäinen H, Myllys V, Skurnik M, Nikkari S (2007) Simultaneous real-time PCR detection of *Bacillus anthracis*, *Francisella tularensis* and *Yersinia pestis*. Eur J Clin Microbiol Infect Dis 26: 207–211.

<https://doi.org/10.1007/s10096-007-0262-z>

Tveten A-K (2013) Prevalence of *Borrelia burgdorferi* sensu stricto, *Borrelia afzelii*, *Borrelia garinii*, and *Borrelia valaisiana* in *Ixodes ricinus* ticks from the northwest of Norway. Scand J Infect Dis 45:681–687.

<https://doi.org/10.3109/00365548.2013.799288>

Vayssier-Taussat M, Moutailler S, Michelet L, Devillers E, Bonnet S, Cheval J, Hébert C, Eloit M (2013) Next generation sequencing uncovers unexpected bacterial pathogens in ticks in Western Europe. PLoS ONE 8:e81439. <https://doi.org/10.1371/journal.pone.0081439>
