# Supplementary material for: One out of ten: low sampling efficiency of cloth dragging challenges abundance estimates of questing ticks
Source: Exp Appl Acarol. 2020 Oct 31;82(4):571–85. doi: 10.1007/s10493-020-00564-5 (PMC7686165; doi:10.1007/s10493-020-00564-5)
Supplement: Supplementary file 2 — Supplementary file2 (DOCX 82 kb) [file 10493_2020_564_MOESM2_ESM.docx]

*Online Resource 2*

**One out of ten: low sampling efficiency of cloth dragging challenges abundance estimates of questing ticks**

Siiri Nyrhilä^1^, Jani J. Sormunen^1,2^, Satu Mäkelä^1^, Ella Sippola^1,2^, Eero J. Vesterinen^2,3^ & Tero Klemola^1^

^1^ Department of Biology, University of Turku, Finland

^2^ Biodiversity Unit, University of Turku, Finland

^3^ Department of Ecology, Swedish University of Agricultural Sciences, Uppsala, Sweden

Corresponding author:

Tero Klemola

Address: Department of Biology, University of Turku, FI-20014 Turku, Finland

Tel.: +358 29 4504216

E-mail: [tero.klemola@utu.fi](mailto:tero.klemola@utu.fi)

*Supplementary material*

**Numbers of caught ticks**

- Numbers of the life stages caught by transect, day and time during the study (Table S1)
- Supplementary graphs depicting interactions of explanatory factors for the count data of caught nymphs in the conducted generalised linear mixed model (Fig. S1)

**Table S1.** Numbers of larvae, nymphs, adult females and adult males caught in cloth-dragging sessions. The dragging in type A transect was conducted once in a sampling session, while the type B transects belonged to the repeated dragging arrangement, in which the dragging was repeated instantly (B_second_) after the first dragging (B_first_)

| **Dragging ID** | **Dragging day: time**  **10–14 June 2019** | **Transect No.: type** | **Larvae (indiv.)** | **Nymphs (indiv.)** | **Females (indiv.)** | **Males (indiv.)** | **Σ Ticks (indiv.)** |
| --- | --- | --- | --- | --- | --- | --- | --- |
| 1 | Mon: morning | A1: A | 0 | 1 | 0 | 0 | 1 |
| 2 | Mon: midday | A1: A | 0 | 2 | 0 | 0 | 2 |
| 3 | Mon: afternoon | A1: A | 0 | 2 | 0 | 0 | 2 |
| 4 | Tue: morning | A1: A | 0 | 1 | 0 | 1 | 2 |
| 5 | Tue: midday | A1: A | 1 | 2 | 0 | 0 | 3 |
| 6 | Tue: afternoon | A1: A | 1 | 0 | 0 | 0 | 1 |
| 7 | Wed: morning | A1: A | 0 | 1 | 0 | 0 | 1 |
| 8 | Wed: midday | A1: A | 0 | 0 | 0 | 0 | 0 |
| 9 | Wed: afternoon | A1: A | 0 | 1 | 0 | 0 | 1 |
| 10 | Thu: morning | A1: A | 0 | 2 | 0 | 0 | 2 |
| 11 | Thu: midday | A1: A | 0 | 0 | 0 | 0 | 0 |
| 12 | Thu: afternoon | A1: A | 0 | 0 | 0 | 0 | 0 |
| 13 | Fri: morning | A1: A | 0 | 0 | 0 | 1 | 1 |
| 14 | Fri: midday | A1: A | 0 | 0 | 1 | 0 | 1 |
| 15 | Fri: afternoon | A1: A | 0 | 0 | 0 | 0 | 0 |
| **Σ All** |  |  | **2** | **12** | **1** | **2** | **17** |

| **Dragging ID** | **Dragging day: time**  **10–14 June 2019** | **Transect No.: type** | **Larvae (indiv.)** | **Nymphs (indiv.)** | **Females (indiv.)** | **Males (indiv.)** | **Σ Ticks (indiv.)** |
| --- | --- | --- | --- | --- | --- | --- | --- |
| 1 | Mon: morning | A2: A | 0 | 2 | 0 | 0 | 2 |
| 2 | Mon: midday | A2: A | 0 | 6 | 1 | 0 | 7 |
| 3 | Mon: afternoon | A2: A | 0 | 4 | 0 | 0 | 4 |
| 4 | Tue: morning | A2: A | 0 | 7 | 0 | 0 | 7 |
| 5 | Tue: midday | A2: A | 1 | 3 | 0 | 0 | 4 |
| 6 | Tue: afternoon | A2: A | 0 | 1 | 0 | 1 | 2 |
| 7 | Wed: morning | A2: A | 1 | 4 | 0 | 1 | 6 |
| 8 | Wed: midday | A2: A | 0 | 0 | 0 | 0 | 0 |
| 9 | Wed: afternoon | A2: A | 0 | 0 | 0 | 0 | 0 |
| 10 | Thu: morning | A2: A | 0 | 4 | 1 | 0 | 5 |
| 11 | Thu: midday | A2: A | 1 | 1 | 0 | 0 | 2 |
| 12 | Thu: afternoon | A2: A | 0 | 2 | 0 | 0 | 2 |
| 13 | Fri: morning | A2: A | 1 | 1 | 0 | 0 | 2 |
| 14 | Fri: midday | A2: A | 2 | 0 | 0 | 0 | 2 |
| 15 | Fri: afternoon | A2: A | 0 | 0 | 0 | 0 | 0 |
| **Σ All** |  |  | **6** | **35** | **2** | **2** | **45** |

| **Dragging ID** | **Dragging day: time**  **10–14 June 2019** | **Transect No.: type** | **Larvae (indiv.)** | **Nymphs (indiv.)** | **Females (indiv.)** | **Males (indiv.)** | **Σ Ticks (indiv.)** |
| --- | --- | --- | --- | --- | --- | --- | --- |
| 1 | Mon: morning | A3: A | 1 | 5 | 0 | 0 | 6 |
| 2 | Mon: midday | A3: A | 0 | 5 | 0 | 0 | 5 |
| 3 | Mon: afternoon | A3: A | 0 | 5 | 0 | 1 | 6 |
| 4 | Tue: morning | A3: A | 0 | 3 | 0 | 1 | 4 |
| 5 | Tue: midday | A3: A | 0 | 2 | 0 | 0 | 2 |
| 6 | Tue: afternoon | A3: A | 3 | 5 | 0 | 0 | 8 |
| 7 | Wed: morning | A3: A | 0 | 6 | 0 | 1 | 7 |
| 8 | Wed: midday | A3: A | 1 | 2 | 0 | 0 | 3 |
| 9 | Wed: afternoon | A3: A | 0 | 4 | 0 | 0 | 4 |
| 10 | Thu: morning | A3: A | 0 | 3 | 1 | 0 | 4 |
| 11 | Thu: midday | A3: A | 0 | 3 | 0 | 1 | 4 |
| 12 | Thu: afternoon | A3: A | 0 | 1 | 1 | 1 | 3 |
| 13 | Fri: morning | A3: A | 1 | 2 | 0 | 0 | 3 |
| 14 | Fri: midday | A3: A | 0 | 0 | 0 | 0 | 0 |
| 15 | Fri: afternoon | A3: A | 6 | 0 | 0 | 0 | 6 |
| **Σ All** |  |  | **12** | **46** | **2** | **5** | **65** |

| **Dragging ID** | **Dragging day: time**  **10–14 June 2019** | **Transect No.: type** | **Larvae (indiv.)** | **Nymphs (indiv.)** | **Females (indiv.)** | **Males (indiv.)** | **Σ Ticks (indiv.)** |
| --- | --- | --- | --- | --- | --- | --- | --- |
| 1 | Mon: morning | A4: A | 1 | 4 | 0 | 0 | 5 |
| 2 | Mon: midday | A4: A | 0 | 7 | 0 | 0 | 7 |
| 3 | Mon: afternoon | A4: A | 0 | 3 | 0 | 0 | 3 |
| 4 | Tue: morning | A4: A | 0 | 5 | 0 | 0 | 5 |
| 5 | Tue: midday | A4: A | 2 | 2 | 0 | 0 | 4 |
| 6 | Tue: afternoon | A4: A | 0 | 1 | 1 | 0 | 2 |
| 7 | Wed: morning | A4: A | 0 | 3 | 0 | 0 | 3 |
| 8 | Wed: midday | A4: A | 0 | 0 | 0 | 0 | 0 |
| 9 | Wed: afternoon | A4: A | 2 | 2 | 0 | 0 | 4 |
| 10 | Thu: morning | A4: A | 4 | 4 | 0 | 1 | 9 |
| 11 | Thu: midday | A4: A | 0 | 1 | 0 | 0 | 1 |
| 12 | Thu: afternoon | A4: A | 0 | 0 | 0 | 1 | 1 |
| 13 | Fri: morning | A4: A | 2 | 0 | 0 | 0 | 2 |
| 14 | Fri: midday | A4: A | 3 | 0 | 0 | 1 | 4 |
| 15 | Fri: afternoon | A4: A | 2 | 0 | 0 | 0 | 2 |
| **Σ All** |  |  | **16** | **32** | **1** | **3** | **52** |

| **Dragging ID** | **Dragging day: time**  **10–14 June 2019** | **Transect No.: type** | **Larvae (indiv.)** | **Nymphs (indiv.)** | **Females (indiv.)** | **Males (indiv.)** | **Σ Ticks (indiv.)** |
| --- | --- | --- | --- | --- | --- | --- | --- |
| 1 | Mon: morning | A5: A | 3 | 4 | 0 | 0 | 7 |
| 2 | Mon: midday | A5: A | 0 | 1 | 0 | 0 | 1 |
| 3 | Mon: afternoon | A5: A | 1 | 1 | 0 | 1 | 3 |
| 4 | Tue: morning | A5: A | 2 | 5 | 0 | 1 | 8 |
| 5 | Tue: midday | A5: A | 8 | 1 | 0 | 0 | 9 |
| 6 | Tue: afternoon | A5: A | 0 | 1 | 0 | 0 | 1 |
| 7 | Wed: morning | A5: A | 1 | 2 | 0 | 0 | 3 |
| 8 | Wed: midday | A5: A | 0 | 0 | 0 | 0 | 0 |
| 9 | Wed: afternoon | A5: A | 0 | 4 | 1 | 0 | 5 |
| 10 | Thu: morning | A5: A | 4 | 2 | 1 | 0 | 7 |
| 11 | Thu: midday | A5: A | 0 | 1 | 0 | 0 | 1 |
| 12 | Thu: afternoon | A5: A | 0 | 3 | 0 | 0 | 3 |
| 13 | Fri: morning | A5: A | 0 | 1 | 0 | 0 | 1 |
| 14 | Fri: midday | A5: A | 0 | 2 | 0 | 0 | 2 |
| 15 | Fri: afternoon | A5: A | 3 | 0 | 0 | 0 | 3 |
| **Σ All** |  |  | **22** | **28** | **2** | **2** | **54** |

| **Dragging ID** | **Dragging day: time**  **10–14 June 2019** | **Transect No.: type** | **Larvae (indiv.)** | **Nymphs (indiv.)** | **Females (indiv.)** | **Males (indiv.)** | **Σ Ticks (indiv.)** |
| --- | --- | --- | --- | --- | --- | --- | --- |
| 1 | Mon: morning | B1: B_first_ | 2 | 0 | 0 | 0 | 2 |
| 2 | Mon: morning | B1: B_second_ | 1 | 1 | 0 | 0 | 2 |
| 3 | Mon: midday | B1: B_first_ | 3 | 2 | 0 | 0 | 5 |
| 4 | Mon: midday | B1: B_second_ | 3 | 0 | 0 | 0 | 3 |
| 5 | Mon: afternoon | B1: B_first_ | 0 | 1 | 0 | 0 | 1 |
| 6 | Mon: afternoon | B1: B_second_ | 0 | 0 | 0 | 0 | 0 |
| 7 | Tue: morning | B1: B_first_ | 0 | 2 | 0 | 1 | 3 |
| 8 | Tue: morning | B1: B_second_ | 0 | 0 | 0 | 0 | 0 |
| 9 | Tue: midday | B1: B_first_ | 1 | 2 | 0 | 0 | 3 |
| 10 | Tue: midday | B1: B_second_ | 0 | 1 | 0 | 0 | 1 |
| 11 | Tue: afternoon | B1: B_first_ | 0 | 2 | 3 | 0 | 5 |
| 12 | Tue: afternoon | B1: B_second_ | 2 | 1 | 0 | 0 | 3 |
| 13 | Wed: morning | B1: B_first_ | 4 | 1 | 1 | 0 | 6 |
| 14 | Wed: morning | B1: B_second_ | 0 | 0 | 0 | 0 | 0 |
| 15 | Wed: midday | B1: B_first_ | 0 | 1 | 0 | 0 | 1 |
| 16 | Wed: midday | B1: B_second_ | 0 | 1 | 0 | 0 | 1 |
| 17 | Wed: afternoon | B1: B_first_ | 1 | 0 | 1 | 1 | 3 |
| 18 | Wed: afternoon | B1: B_second_ | 0 | 0 | 1 | 0 | 1 |
| 19 | Thu: morning | B1: B_first_ | 1 | 0 | 0 | 1 | 2 |
| 20 | Thu: morning | B1: B_second_ | 0 | 0 | 0 | 0 | 0 |
| 21 | Thu: midday | B1: B_first_ | 0 | 0 | 0 | 0 | 0 |
| 22 | Thu: midday | B1: B_second_ | 0 | 0 | 0 | 0 | 0 |
| 23 | Thu: afternoon | B1: B_first_ | 0 | 2 | 1 | 0 | 3 |
| 24 | Thu: afternoon | B1: B_second_ | 2 | 0 | 0 | 0 | 2 |
| 25 | Fri: morning | B1: B_first_ | 0 | 1 | 0 | 0 | 1 |
| 26 | Fri: morning | B1: B_second_ | 0 | 0 | 0 | 0 | 0 |
| 27 | Fri: midday | B1: B_first_ | 0 | 1 | 1 | 0 | 2 |
| 28 | Fri: midday | B1: B_second_ | 2 | 0 | 0 | 0 | 2 |
| 29 | Fri: afternoon | B1: B_first_ | 0 | 0 | 0 | 0 | 0 |
| 30 | Fri: afternoon | B1: B_second_ | 0 | 0 | 0 | 0 | 0 |
| **Σ All** |  |  | **22** | **19** | **8** | **3** | **52** |

| **Dragging ID** | **Dragging day: time**  **10–14 June 2019** | **Transect No.: type** | **Larvae (indiv.)** | **Nymphs (indiv.)** | **Females (indiv.)** | **Males (indiv.)** | **Σ Ticks (indiv.)** |
| --- | --- | --- | --- | --- | --- | --- | --- |
| 1 | Mon: morning | B2: B_first_ | 0 | 1 | 0 | 0 | 1 |
| 2 | Mon: morning | B2: B_second_ | 2 | 0 | 0 | 0 | 2 |
| 3 | Mon: midday | B2: B_first_ | 0 | 0 | 0 | 0 | 0 |
| 4 | Mon: midday | B2: B_second_ | 0 | 0 | 0 | 0 | 0 |
| 5 | Mon: afternoon | B2: B_first_ | 0 | 0 | 0 | 0 | 0 |
| 6 | Mon: afternoon | B2: B_second_ | 0 | 0 | 0 | 0 | 0 |
| 7 | Tue: morning | B2: B_first_ | 0 | 4 | 0 | 0 | 4 |
| 8 | Tue: morning | B2: B_second_ | 0 | 0 | 1 | 0 | 1 |
| 9 | Tue: midday | B2: B_first_ | 0 | 2 | 0 | 0 | 2 |
| 10 | Tue: midday | B2: B_second_ | 0 | 0 | 0 | 0 | 0 |
| 11 | Tue: afternoon | B2: B_first_ | 0 | 0 | 0 | 0 | 0 |
| 12 | Tue: afternoon | B2: B_second_ | 0 | 0 | 0 | 0 | 0 |
| 13 | Wed: morning | B2: B_first_ | 0 | 3 | 0 | 0 | 3 |
| 14 | Wed: morning | B2: B_second_ | 0 | 0 | 0 | 0 | 0 |
| 15 | Wed: midday | B2: B_first_ | 0 | 0 | 0 | 0 | 0 |
| 16 | Wed: midday | B2: B_second_ | 0 | 1 | 0 | 0 | 1 |
| 17 | Wed: afternoon | B2: B_first_ | 0 | 3 | 0 | 0 | 3 |
| 18 | Wed: afternoon | B2: B_second_ | 0 | 0 | 0 | 0 | 0 |
| 19 | Thu: morning | B2: B_first_ | 0 | 1 | 0 | 0 | 1 |
| 20 | Thu: morning | B2: B_second_ | 0 | 0 | 0 | 0 | 0 |
| 21 | Thu: midday | B2: B_first_ | 1 | 0 | 0 | 0 | 1 |
| 22 | Thu: midday | B2: B_second_ | 0 | 0 | 0 | 0 | 0 |
| 23 | Thu: afternoon | B2: B_first_ | 0 | 1 | 0 | 0 | 1 |
| 24 | Thu: afternoon | B2: B_second_ | 1 | 0 | 0 | 0 | 1 |
| 25 | Fri: morning | B2: B_first_ | 4 | 0 | 1 | 0 | 5 |
| 26 | Fri: morning | B2: B_second_ | 0 | 1 | 0 | 0 | 1 |
| 27 | Fri: midday | B2: B_first_ | 3 | 0 | 0 | 1 | 4 |
| 28 | Fri: midday | B2: B_second_ | 0 | 0 | 0 | 0 | 0 |
| 29 | Fri: afternoon | B2: B_first_ | 4 | 0 | 0 | 0 | 4 |
| 30 | Fri: afternoon | B2: B_second_ | 1 | 0 | 1 | 0 | 2 |
| **Σ All** |  |  | **16** | **17** | **3** | **1** | **37** |

| **Dragging ID** | **Dragging day: time**  **10–14 June 2019** | **Transect No.: type** | **Larvae (indiv.)** | **Nymphs (indiv.)** | **Females (indiv.)** | **Males (indiv.)** | **Σ Ticks (indiv.)** |
| --- | --- | --- | --- | --- | --- | --- | --- |
| 1 | Mon: morning | B3: B_first_ | 0 | 2 | 0 | 1 | 3 |
| 2 | Mon: morning | B3: B_second_ | 0 | 0 | 0 | 0 | 0 |
| 3 | Mon: midday | B3: B_first_ | 0 | 1 | 0 | 0 | 1 |
| 4 | Mon: midday | B3: B_second_ | 0 | 0 | 0 | 0 | 0 |
| 5 | Mon: afternoon | B3: B_first_ | 0 | 1 | 0 | 0 | 1 |
| 6 | Mon: afternoon | B3: B_second_ | 3 | 0 | 0 | 0 | 3 |
| 7 | Tue: morning | B3: B_first_ | 0 | 3 | 1 | 1 | 5 |
| 8 | Tue: morning | B3: B_second_ | 3 | 0 | 0 | 0 | 3 |
| 9 | Tue: midday | B3: B_first_ | 6 | 1 | 0 | 0 | 7 |
| 10 | Tue: midday | B3: B_second_ | 0 | 1 | 0 | 0 | 1 |
| 11 | Tue: afternoon | B3: B_first_ | 0 | 1 | 0 | 0 | 1 |
| 12 | Tue: afternoon | B3: B_second_ | 0 | 1 | 0 | 0 | 1 |
| 13 | Wed: morning | B3: B_first_ | 6 | 4 | 0 | 0 | 10 |
| 14 | Wed: morning | B3: B_second_ | 2 | 0 | 0 | 0 | 2 |
| 15 | Wed: midday | B3: B_first_ | 2 | 1 | 0 | 0 | 3 |
| 16 | Wed: midday | B3: B_second_ | 3 | 0 | 0 | 0 | 3 |
| 17 | Wed: afternoon | B3: B_first_ | 2 | 0 | 0 | 0 | 2 |
| 18 | Wed: afternoon | B3: B_second_ | 0 | 1 | 1 | 0 | 2 |
| 19 | Thu: morning | B3: B_first_ | 7 | 3 | 0 | 0 | 10 |
| 20 | Thu: morning | B3: B_second_ | 0 | 1 | 0 | 0 | 1 |
| 21 | Thu: midday | B3: B_first_ | 2 | 0 | 0 | 0 | 2 |
| 22 | Thu: midday | B3: B_second_ | 0 | 0 | 1 | 0 | 1 |
| 23 | Thu: afternoon | B3: B_first_ | 6 | 1 | 0 | 0 | 7 |
| 24 | Thu: afternoon | B3: B_second_ | 0 | 0 | 0 | 0 | 0 |
| 25 | Fri: morning | B3: B_first_ | 0 | 0 | 0 | 0 | 0 |
| 26 | Fri: morning | B3: B_second_ | 0 | 0 | 0 | 0 | 0 |
| 27 | Fri: midday | B3: B_first_ | 0 | 0 | 0 | 0 | 0 |
| 28 | Fri: midday | B3: B_second_ | 0 | 0 | 0 | 0 | 0 |
| 29 | Fri: afternoon | B3: B_first_ | 0 | 0 | 0 | 0 | 0 |
| 30 | Fri: afternoon | B3: B_second_ | 2 | 0 | 0 | 0 | 2 |
| **Σ All** |  |  | **44** | **22** | **3** | **2** | **71** |

| **Dragging ID** | **Dragging day: time**  **10–14 June 2019** | **Transect No.: type** | **Larvae (indiv.)** | **Nymphs (indiv.)** | **Females (indiv.)** | **Males (indiv.)** | **Σ Ticks (indiv.)** |
| --- | --- | --- | --- | --- | --- | --- | --- |
| 1 | Mon: morning | B4: B_first_ | 0 | 4 | 0 | 0 | 4 |
| 2 | Mon: morning | B4: B_second_ | 0 | 1 | 0 | 0 | 1 |
| 3 | Mon: midday | B4: B_first_ | 0 | 1 | 1 | 0 | 2 |
| 4 | Mon: midday | B4: B_second_ | 0 | 0 | 0 | 0 | 0 |
| 5 | Mon: afternoon | B4: B_first_ | 0 | 2 | 0 | 0 | 2 |
| 6 | Mon: afternoon | B4: B_second_ | 0 | 1 | 0 | 0 | 1 |
| 7 | Tue: morning | B4: B_first_ | 4 | 3 | 0 | 0 | 7 |
| 8 | Tue: morning | B4: B_second_ | 0 | 2 | 0 | 0 | 2 |
| 9 | Tue: midday | B4: B_first_ | 2 | 3 | 0 | 1 | 6 |
| 10 | Tue: midday | B4: B_second_ | 1 | 1 | 0 | 0 | 2 |
| 11 | Tue: afternoon | B4: B_first_ | 2 | 2 | 1 | 0 | 5 |
| 12 | Tue: afternoon | B4: B_second_ | 0 | 0 | 0 | 0 | 0 |
| 13 | Wed: morning | B4: B_first_ | 2 | 2 | 1 | 1 | 6 |
| 14 | Wed: morning | B4: B_second_ | 0 | 1 | 0 | 0 | 1 |
| 15 | Wed: midday | B4: B_first_ | 0 | 3 | 0 | 0 | 3 |
| 16 | Wed: midday | B4: B_second_ | 0 | 0 | 0 | 0 | 0 |
| 17 | Wed: afternoon | B4: B_first_ | 1 | 2 | 0 | 0 | 3 |
| 18 | Wed: afternoon | B4: B_second_ | 0 | 1 | 0 | 0 | 1 |
| 19 | Thu: morning | B4: B_first_ | 4 | 1 | 0 | 0 | 5 |
| 20 | Thu: morning | B4: B_second_ | 1 | 1 | 0 | 0 | 2 |
| 21 | Thu: midday | B4: B_first_ | 0 | 0 | 0 | 1 | 1 |
| 22 | Thu: midday | B4: B_second_ | 0 | 0 | 0 | 0 | 0 |
| 23 | Thu: afternoon | B4: B_first_ | 0 | 1 | 0 | 0 | 1 |
| 24 | Thu: afternoon | B4: B_second_ | 0 | 0 | 0 | 0 | 0 |
| 25 | Fri: morning | B4: B_first_ | 4 | 2 | 1 | 0 | 7 |
| 26 | Fri: morning | B4: B_second_ | 0 | 0 | 0 | 0 | 0 |
| 27 | Fri: midday | B4: B_first_ | 6 | 1 | 0 | 0 | 7 |
| 28 | Fri: midday | B4: B_second_ | 1 | 1 | 0 | 0 | 2 |
| 29 | Fri: afternoon | B4: B_first_ | 8 | 1 | 0 | 0 | 9 |
| 30 | Fri: afternoon | B4: B_second_ | 0 | 0 | 0 | 0 | 0 |
| **Σ All** |  |  | **36** | **37** | **4** | **3** | **80** |

| **Dragging ID** | **Dragging day: time**  **10–14 June 2019** | **Transect No.: type** | **Larvae (indiv.)** | **Nymphs (indiv.)** | **Females (indiv.)** | **Males (indiv.)** | **Σ Ticks (indiv.)** |
| --- | --- | --- | --- | --- | --- | --- | --- |
| 1 | Mon: morning | B5: B_first_ | 0 | 0 | 0 | 0 | 0 |
| 2 | Mon: morning | B5: B_second_ | 0 | 1 | 0 | 0 | 1 |
| 3 | Mon: midday | B5: B_first_ | 0 | 1 | 1 | 0 | 2 |
| 4 | Mon: midday | B5: B_second_ | 0 | 1 | 1 | 0 | 2 |
| 5 | Mon: afternoon | B5: B_first_ | 0 | 3 | 1 | 0 | 4 |
| 6 | Mon: afternoon | B5: B_second_ | 0 | 1 | 0 | 0 | 1 |
| 7 | Tue: morning | B5: B_first_ | 0 | 2 | 1 | 0 | 3 |
| 8 | Tue: morning | B5: B_second_ | 0 | 0 | 1 | 0 | 1 |
| 9 | Tue: midday | B5: B_first_ | 0 | 2 | 0 | 0 | 2 |
| 10 | Tue: midday | B5: B_second_ | 0 | 2 | 0 | 0 | 2 |
| 11 | Tue: afternoon | B5: B_first_ | 0 | 2 | 0 | 0 | 2 |
| 12 | Tue: afternoon | B5: B_second_ | 0 | 0 | 0 | 0 | 0 |
| 13 | Wed: morning | B5: B_first_ | 0 | 2 | 0 | 1 | 3 |
| 14 | Wed: morning | B5: B_second_ | 0 | 0 | 0 | 0 | 0 |
| 15 | Wed: midday | B5: B_first_ | 0 | 2 | 0 | 0 | 2 |
| 16 | Wed: midday | B5: B_second_ | 0 | 0 | 0 | 0 | 0 |
| 17 | Wed: afternoon | B5: B_first_ | 0 | 0 | 0 | 0 | 0 |
| 18 | Wed: afternoon | B5: B_second_ | 0 | 2 | 0 | 0 | 2 |
| 19 | Thu: morning | B5: B_first_ | 1 | 0 | 0 | 1 | 2 |
| 20 | Thu: morning | B5: B_second_ | 0 | 0 | 0 | 0 | 0 |
| 21 | Thu: midday | B5: B_first_ | 0 | 1 | 0 | 0 | 1 |
| 22 | Thu: midday | B5: B_second_ | 0 | 1 | 0 | 0 | 1 |
| 23 | Thu: afternoon | B5: B_first_ | 0 | 0 | 0 | 0 | 0 |
| 24 | Thu: afternoon | B5: B_second_ | 0 | 0 | 0 | 0 | 0 |
| 25 | Fri: morning | B5: B_first_ | 0 | 2 | 0 | 0 | 2 |
| 26 | Fri: morning | B5: B_second_ | 0 | 0 | 1 | 0 | 1 |
| 27 | Fri: midday | B5: B_first_ | 0 | 1 | 0 | 0 | 1 |
| 28 | Fri: midday | B5: B_second_ | 0 | 0 | 0 | 0 | 0 |
| 29 | Fri: afternoon | B5: B_first_ | 0 | 0 | 0 | 0 | 0 |
| 30 | Fri: afternoon | B5: B_second_ | 0 | 0 | 0 | 0 | 0 |
| **Σ All** |  |  | **1** | **26** | **6** | **2** | **35** |

**Fig S1.** Model-derived least-squares mean (with 95 % confidence intervals) estimates of caught nymphs (individuals per 10-m cloth dragging) from the GLMM with negative binomial error distribution. Estimates depict interactions ‘Sampling method’ × ‘Sampling day’ (a) and ‘Sampling time’ × ‘Sampling day’ (b). See Table 1 in the main text for GLMM statistics. In (a), A, B_first_ and B_second_ refer to types of sampling transects. Dragging in type A transect was conducted once in a sampling session, while the type B transects belonged to the repeated dragging arrangement, in which the dragging was repeated instantly (B_second_) after the first dragging (B_first_)
