# Supplementary material for: One out of ten: low sampling efficiency of cloth dragging challenges abundance estimates of questing ticks
Source: Exp Appl Acarol. 2020 Oct 31;82(4):571–85. doi: 10.1007/s10493-020-00564-5 (PMC7686165; doi:10.1007/s10493-020-00564-5)
Supplement: Supplementary file 3 — Supplementary file3 (DOCX 18 kb) [file 10493_2020_564_MOESM3_ESM.docx]

*Online Resource 3*

**One out of ten: low sampling efficiency of cloth dragging challenges abundance estimates of questing ticks**

Siiri Nyrhilä^1^, Jani J. Sormunen^1,2^, Satu Mäkelä^1^, Ella Sippola^1,2^, Eero J. Vesterinen^2,3^ & Tero Klemola^1^

^1^ Department of Biology, University of Turku, Finland

^2^ Biodiversity Unit, University of Turku, Finland

^3^ Department of Ecology, Swedish University of Agricultural Sciences, Uppsala, Sweden

Corresponding author:

Tero Klemola

Address: Department of Biology, University of Turku, FI-20014 Turku, Finland

Tel.: +358 29 4504216

E-mail: [tero.klemola@utu.fi](mailto:tero.klemola@utu.fi)

*Supplementary material*

**Generalised linear mixed model analysis of the pathogen data**

**Table S1.** Model-derived probability estimates (least-squares means [95 % CI]) of fixed effects from binomial GLMMs for detecting a pathogen positive tick sample in the cloth dragging

–––––––––––––––––––––––––––––––––––––––––––––––––––––––––––––––––––––––––––––––––––––––

Effect Class level Any pathogen *B. burgdorferi* s.l.

–––––––––––––––––––––––––––––––––––––––––––––––––––––––––––––––––––––––––––––––––––––––

Sampling method^a^ Transect A 0.33 [0.22–0.45] 0.19 [0.12–0.29]

Transect B_first_ 0.45 [0.33–0.59] 0.30 [0.20–0.43]

Transect B_second_ 0.35 [0.20–0.54] 0.22 [0.10–0.40]

Sampling time Morning 0.36 [0.26–0.48] 0.25 [0.17–0.36]

Midday 0.35 [0.24–0.48] 0.20 [0.12–0.31]

Afternoon 0.42 [0.29–0.55] 0.25 [0.16–0.38]

Sampling day Monday 0.37 [0.25–0.51] 0.26 [0.16–0.38]

10–14 June 2019 Tuesday 0.35 [0.24–0.48] 0.26 [0.17–0.39]

Wednesday 0.40 [0.27–0.54] 0.27 [0.17–0.40]

Thursday 0.39 [0.25–0.55] 0.27 [0.15–0.42]

Friday 0.37 [0.20–0.58] 0.14 [0.05–0.33]

–––––––––––––––––––––––––––––––––––––––––––––––––––––––––––––––––––––––––––––––––––––––

^a^ Transects A, B_first_ and B_second_ refer to types of cloth-dragging transects. The dragging in type A transects was conducted once in a sampling session, while the type B transects belonged to the repeated dragging arrangement, in which the dragging was repeated instantly (B_second_) after the first dragging (B_first_)
